# Genetic Polymorphism rs6505162 in MicroRNA-423 May Not Be Associated with Susceptibility of Breast Cancer: A Systematic Review and Meta-Analysis

**DOI:** 10.1155/2021/3003951

**Published:** 2021-11-26

**Authors:** Zhi Li, Jin Wang, Hui-bing Chen, Xiao-Mei Guo, Xiao-Ping Chen, Meng Wang, Li-Juan Dong, Min-Min Zhang

**Affiliations:** ^1^Department of Nursing, Zhongshan Hospital of Chinese Traditional Medicine, Zhongshan 528400, China; ^2^Department of Nephrology, Zhongshan Hospital of Chinese Traditional Medicine, Zhongshan 528400, China; ^3^Department of Anorectal, Zhongshan Hospital of Chinese Traditional Medicine, Zhongshan 528400, China; ^4^Department of Oncology, Zhongshan Hospital of Chinese Traditional Medicine, Zhongshan 528400, China; ^5^Department of Thyroid and Breast Surgery, Liuzhou People's Hospital, Liuzhou 545005, China

## Abstract

**Background:**

MicroRNA-423 (miR-423) rs6505162 polymorphism is found to be associated with breast cancer (BC) risk. However, the results were inconsistent. This study meta-analyzed the literature on possible association between rs6505162 polymorphism and BC risk.

**Methods:**

PubMed, Embase, Google Scholar, and the Chinese National Knowledge Infrastructure (CNKI) databases were systematically searched to identify relevant studies. Meta-analyses were performed to examine the association between rs6505162 polymorphism and BC.

**Results:**

None of the five genetic models suggested a significant association between rs6505162 polymorphism and BC risk: allelic model, OR 1.02, 95% CI 0.18–1.28, *P*=0.85; recessive model, OR 0.99, 95% CI 0.72–1.38, *P*=0.97; dominant model, OR 0.93, 95% CI 0.72–1.21, *P*=0.60; homozygous model, OR 1.04, 95% CI 0.66–1.65, *P*=0.87; and heterozygous model, OR 1.07, 95% CI 0.90–1.28, *P*=0.45. Similar results were obtained in subgroup analyses of Asian, Chinese, and Caucasian patients.

**Conclusion:**

The available evidence suggests no significant association between rs6505162 polymorphism and BC risk. These conclusions should be verified in large, well-designed studies.

## 1. Introduction

Breast cancer (BC) continues to disrupt the lives of millions of women. For many years, BC has consistently ranked among the top cancers in the women, both in terms of incidence and mortality [1]. As we all know, age, menstrual status (early menarche age and delayed menopause), reproduction (late age at first birth), genetic predisposition (higher incidence among close family members and first degree relatives in the breast cancer patients), lifestyle (saturated fat diet, alcohol excessive intake, and obesity), and so on are generally considered to be the causes of BC [2]. However, most causes of BC are not yet clearly understood. Genetic factors have been reported to play an important role in BC development. For instance, mutation in BRCA1 and BRCA2 and low-penetrance common genetic variants were identified as breast cancer risk factors [3]. Recent research studies have shown that one miRNA can potentially affect the expression of many genes to various degrees, and it could participate in the control of numerous metabolic pathways, including cellular growth and differentiation, suggesting that single nucleotide polymorphisms located within miRNAs can have extremely far reaching effects and may affect the development of multiple diseases, including BC [4–8]. These results indicated that miRNAs may also be risk factors for BC.

miR-423 is located in frequently amplified region of chromosome 17q11.2 and can produce two mature sequences: miR-423-3p and miR-423-5p [6]. Recent studies have shown that rs6505162: C > A, in pre-miR-423 increases risk of familial BC in families with a strong history of BC [7] and SNP rs6505162 in pre-miR-423 affects the mature miR expression, and then miR-423 may play a oncogenic role in breast tumorigenesis [8]. However, results of a recent meta-analysis including only two case-control studies on rs6505162 showed no relationship between rs6505162 polymorphism and BC risk [9]. Given the limited sample size, there is currently no consensus on whether there exists an association between rs6505162 polymorphism and BC risk.

As some new studies published, we conducted this meta-analysis of all relevant literatures to provide comprehensive and reliable insights. To the best of our knowledge, this is the first meta-analysis especially concerning rs6505162 polymorphism and BC risk, and it has the largest sample at present, compared with those published ones.

## 2. Materials and Methods

### 2.1. Search Strategy

All clinical and experimental case-control studies of polymorphisms in the miR-423 gene and BC published through May 15, 2021, were identified through systematic searches in PubMed, Embase, Google Scholar, and the Chinese National Knowledge Infrastructure (CNKI) databases, without language restrictions. The search terms used were: *microRNA-423; miR-423; rs6505162;* these three terms in combination with *polymorphism, polymorphisms, SNP, variant, variants, variation, genotype, genetic*, or *mutation;* and all of the above terms in combination with *breast cancer, mammary cancer*, *or mammary adenocarcinoma*. Reference lists in identified articles and reviews were also searched manually to identify additional eligible studies.

### 2.2. Inclusion Criteria

To be included in our review and meta-analysis, studies had to (1) have a case-control design for assessing the association between rs6505162 polymorphism and BC risk; (2) be accessible as a full-text article and report sufficient data for estimating odds ratios (ORs) with 95% confidence intervals (CIs); (3) report genotype frequencies; and (4) involve humans rather than animal models.

### 2.3. Data Extraction

Two authors (ZL and LJD) independently extracted the following data from included studies: first author's family name, year of publication, ethnicity, testing methods, NOS score, *P* value for Hardy–Weinberg equilibrium (HWE) in controls, control source, sample size, matched parameters, and numbers and genotypes of cases and controls. Discrepancies were resolved by consensus. Only those studies that met the predetermined inclusion criteria were included.

### 2.4. Assessment of Methodological Quality

To assess the quality of the studies included in this analysis, the Newcastle–Ottawa Scale was applied independently by two assessors (ZL and LJD) [10] (Table 1). On the 10-point Newcastle–Ottawa Scale, scores of 5–9 points (stars) are considered to indicate generally high methodological quality, while scores of 0–4 stars are considered to indicate poor quality [11]. Any disagreements about Newcastle–Ottawa scores were resolved by other authors following a comprehensive reassessment. Only high-quality studies were included in the meta-analysis.

### 2.5. Statistical Analysis

Unadjusted odds ratios (ORs) with 95% confidence intervals (CIs) were used to assess the strength of the association between rs6505162 polymorphism and BC risk based on genotype frequencies in cases and controls. The significance of pooled ORs was determined using the *Z* test, with *P* < 0.05 defined as the significance threshold. Meta-analysis was conducted using a fixed-effect model when *P* > 0.10 for the *Q* test, indicating lack of heterogeneity among studies; otherwise, a random-effect model was used. All these statistical tests were performed using Review Manager 5.3 (Cochrane Collaboration).

Publication bias was assessed using Begg's funnel plots and Egger's weighted regression in Stata 12.0 (Stata Corp., College Station, TX, USA), with *P* < 0.05 considered statistically significant.

## 3. Results

### 3.1. Description of Studies

**Figure 1:**
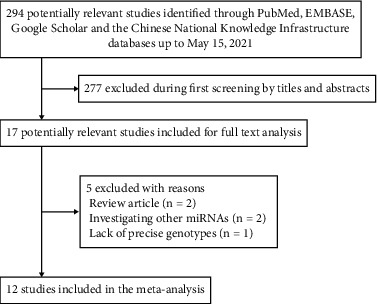
Flowchart showing search strategies, selection criteria, and included studies.

Figure 1 shows a flowchart illustrating the process of searching for and selecting studies. A total of 294 potentially relevant publications were identified. Of these, we excluded 277 studies during initial screening based on review of the titles and abstracts. During analysis of the full text of the remaining articles, two studies were excluded for investigating other miRNAs [12, 13], two studies were excluded because they were review articles [14, 15], and one study was excluded because it did not report precise genotypes [16].

In the end, 12 studies [7, 17–27] were included in this meta-analysis based on our search strategy and inclusion criteria. Their characteristics and genotype distributions are summarized in Tables 1 and 2, respectively. The distribution of genotypes in controls was consistent with Hardy–Weinberg equilibrium (HWE, *P* > 0.05) in all but one study [25]. The overall quality of the included studies was adequate, and the mean Newcastle–Ottawa score for the included studies was 6.75 (Table 3).

### 3.2. Quantitative Data Synthesis

**Figure 2:**
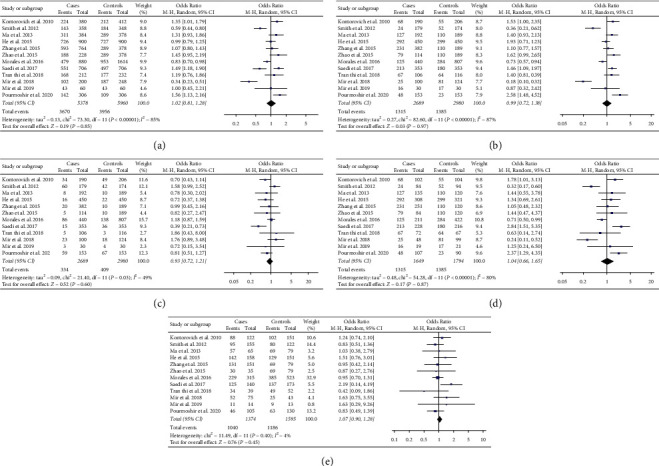
Forest plot showing the relationship between microRNA-423 rs6505162 polymorphism and breast cancer risk in total population according to different genetic models: (a) allelic model (G-allele vs. A-allele), (b) recessive model (GG vs. AG + AA), (c) dominant model (AA vs. AG + GG), (d) homozygous model (GG vs. AA), and (e) heterozygous model (AG vs. AA). Abbreviations: CI, confidence interval; d*f*, degree of freedom; MH, Mantel–Haenszel.

The meta-analysis of a possible association between rs6505162 polymorphism and BC risk is summarized in Table 4. Based on the total study population including 2,689 cases and 2,980 controls from 12 studies [7, 17–27], none of the five genetic models indicated a significant association: allelic model, OR 1.02, 95% CI 0.18–1.28, *P*=0.85 (Figure 2(a)); recessive model, OR 0.99, 95% CI 0.72–1.38, *P*=0.97 (Figure 2(b)); dominant model, OR 0.93, 95% CI 0.72–1.21, *P*=0.60 (Figure 2(c)); homozygous model, OR 1.04, 95% CI 0.66–1.65, *P*=0.87 (Figure 2(d)); and heterozygous model, OR 1.07, 95% CI 0.90–1.28, *P*=0.45 (Figure 2(e)).

Next we meta-analyzed data for subgroups based on ethnicity. Meta-analysis of 9 studies [19–27] involving 1,880 Asian cases and 1,793 Asian controls showed no evidence of a significant association rs6505162 polymorphism and BC risk in any of the five genetic models (Table 4): allelic model, OR = 1.09, 95% CI 0.82–1.44, *P*=0.56; recessive model, OR = 1.10, 95% CI = 0.75–1.61, *P*=0.64; dominant model, OR = 0.81, 95% CI = 0.63–1.03, *P*=0.09; homozygous model, OR = 1.20, 95% CI = 0.69–2.08, *P*=0.52; and heterozygous model, OR = 1.20, 95% CI = 0.92–1.56, *P*=0.18.

Similarly, no evidence of an association was identified in meta-analysis of 4 studies [19–22] involving 1,138 Chinese cases and 1,017 Chinese controls (Table 4): allelic model, OR = 1.12, 95% CI = 0.97–1.30, *P*=0.13; recessive model, OR = 1.13, 95% CI = 0.95–1.35, *P*=0.18; dominant model, OR = 0.81, 95% CI = 0.54–1.22, *P*=0.32; homozygous model, OR = 1.29, 95% CI = 0.85–1.95, *P*=0.24; and heterozygous model, OR = 1.15, 95% CI = 0.75–1.76, *P*=0.53.

Also, no evidence of an association was identified in meta-analysis of 3 studies [7, 17, 18] involving 809 Caucasian cases and 1,187 Chinese controls (Table 4): allelic model, OR = 0.87, 95% CI = 0.58–1.31, *P*=0.51; recessive model, OR = 0.75, 95% CI = 0.38–1.48, *P*=0.41; dominant model, OR = 1.11, 95% CI = 0.74–1.66, *P*=0.63; homozygous model, OR = 0.75, 95% CI = 0.33–1.70, *P*=0.49; and heterozygous model, OR = 0.98, 95% CI = 0.77–1.24, *P*=0.84.

Lastly, no evidence of an association was identified in meta-analysis of 5 studies [18–21, 27] involving 1,356 female cases and 1,155 female controls (Table 4): allelic model, OR = 1.05, 95% CI = 0.77–1.42, *P*=0.78; recessive model, OR = 1.06, 95% CI = 0.66–1.71, *P*=0.81; dominant model, OR = 1.00, 95% CI = 0.77–1.30, *P*=0.99; homozygous model, OR = 1.08, 95% CI = 0.52–2.27, *P*=0.83; and heterozygous model, OR = 0.95, 95% CI = 0.72–1.26, *P*=0.73.

### 3.3. Sensitivity Analysis

The robustness of the meta-analysis of 12 studies examining a possible association between rs6505162 polymorphism and BC risk was assessed by repeating the meta-analysis after excluding a study [25] in which the *P* value associated with HWE was less than 0.05. Deleting these data from the meta-analysis did not alter the results obtained using any of the five genetic models, whether for the entire study population or the Asian population.

### 3.4. Publication Bias

**Figure 3:**
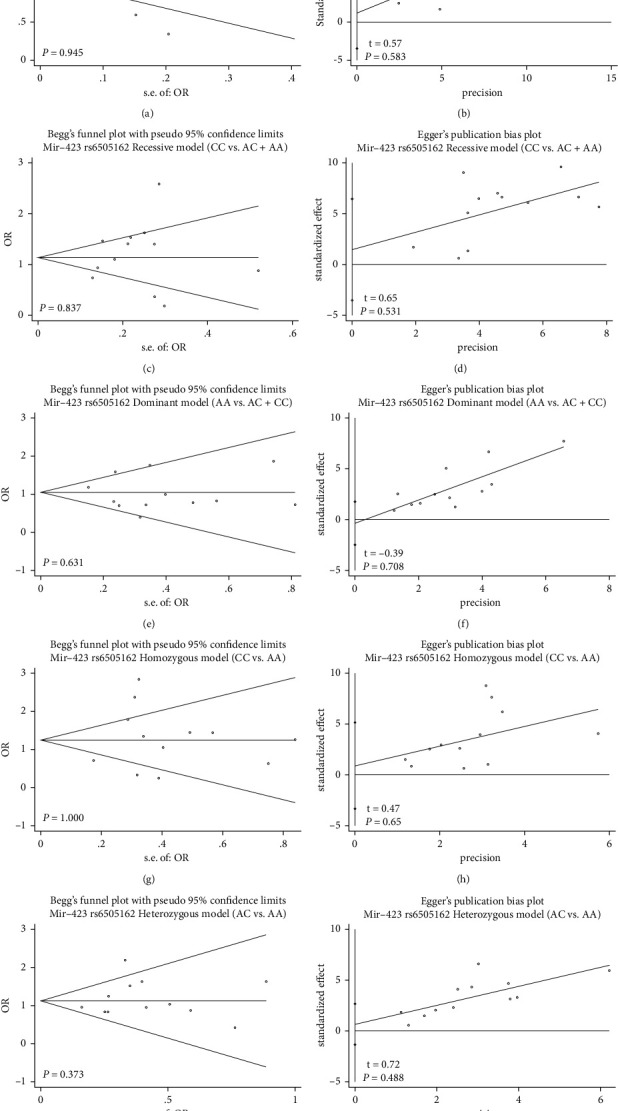
Begg's funnel plot (a) and Egger's test (b) to assess publication bias risk in analysis of the association between microRNA-423 rs6505162 polymorphism and breast cancer risk in total population according to all the genotype models.

Potential publication bias in this meta-analysis was assessed using Begg's funnel plot and Egger's test. In any of the five genetic models, respectively, no obvious asymmetry was observed in Begg's funnel plots (Figures 3(a), 3(c), 3(e), 3(g), and 3(i)) and Egger's test of rs6505162 polymorphism (Figures 3(b), 3(d), 3(f), 3(h), and 3(j)). *P* values for Begg's funnel plots and Egger's tests were all greater than 0.05. These results suggest no potential publication bias.

## 4. Discussion

In order to investigate the relationship between rs6505162 polymorphism and BC risk, a few recent meta-analyses [9, 28–30] have reported their findings. However, their results were inconsistent. Meta-analysis by Chen et al. [28] with 16 case-control studies included suggested that rs6505162 polymorphism might be associated with a reduced risk of cancers but not with BC risk in subgroup analysis of 5 case-control studies. Meta-analysis by Zhang et al. [29] with 6 case-control studies included suggested that a significantly decreased cancer risk was observed in lung cancer for rs6505162 but not in BC risk. Meta-analysis by Li et al. [30] with 8 case-control studies included suggested rs6505162 decreases the risk of cancer, showing that it is the protective factor of cancer. But subgroup analysis for BC risk was not performed.

Those previous meta-analyses did not specially focus on BC, much less on BC by subgroup analysis by ethnicity. In order to evaluate available evidence on the possible association between rs6505162 polymorphism in miR-423 promoter and BC risk, a more detailed meta-analysis was performed. Results showed that miR-423 rs6505162 might not be associated with BC risk, regardless of ethnicity. Even though our results supported previous studies [28, 29], given larger sample with 12 case-control studies included, ours should be more convincing.

Although null results were obtained in the current study on rs6505162 polymorphism with larger sample, we really hope they would provide a reference for future studies. Nonetheless, the work still has several limitations that may affect interpretation of the results. Firstly, the *P* value for HWE in one study [25] was less than 0.05, making these study populations not being representative of the broader target population. Nevertheless, sensitivity analyses showed that deleting the study did not alter the results. Secondly, the studies may be subject to performance bias, attrition bias, and reporting bias, although Newcastle–Ottawa scores were more than 5 for all studies, indicating high quality. Thirdly, additional confounding factors such as age, gender, and tumor status may affect the results. In order to reduce the effect of those confounding factors above on the results, we have tried our best to make stratified analysis based on those factors. In the end, only gender could be taken into account. A total of 5 case-control studies [18–21, 27] of which the patients were all definitely female were selected to investigate the relationship between rs6505162 polymorphism and BC risk on females. Nevertheless, these studies either did not report age and tumor status or aggregated them in different ways, resulting in a failure to include them in the meta-analysis. Lastly, methods used to test for polymorphisms were not uniform and they varied in sensitivity and specificity, which may reduce the robustness of the meta-analysis.

In conclusion, this study performed an extensive assessment based on a larger sample size than the previous pooled analysis and suggested no significant association between miR-423 rs6505162 polymorphism and BC risk. These conclusions should be verified in large, well-designed studies.

## Figures and Tables

**Table 1:** Characteristics of studies included in the meta-analysis.

First author	Year	Ethnicity	Country	Cancer type	Testing method	NOS score	*P* for HWE	Control source	Sample size (*n*)		Matched parameters
									Cases	Controls	
Kontorovich et al. [17]	2010	Caucasian	Israel	BRCA1, BRCA2	iPLEX	6	0.899	PB	190	206	Undetermined
Smith et al. [18]	2012	Caucasian	Australia	—	HRM	7	0.307	HB	179	174	Age, sex, ethnicity
Ma et al. [19]	2013	Asian	China	TNBC	MassArray	7	0.847	HB	192	189	Age, sex, ethnicity, smoking status
He et al. [20]	2015	Asian	China	—	MassArray	8	0.103	PB	450	450	Age, menopausal status
Zhang et al. [21]	2015	Asian	China	—	MassArray	8	0.847	PB	382	189	Age, smoking status
Zhao et al. [22]	2015	Asian	China	—	Sequencing	6	0.847	PB	114	189	Undetermined
Morales et al. [7]	2016	Caucasian	Chile	—	TaqMan	6	0.700	HB	440	807	Age, socioeconomic strata
Saedi et al. [23]	2017	Asian	Iran	—	PCR-RFLP	6	0.196	HB	353	353	Undetermined
Tran Thi et al. [24]	2018	Asian	Vietnam	—	HRM	6	0.071	PB	106	116	Undetermined
Mir et al. [25]	2018	Asian	Saudi Arabia	—	ARMS-PCR	7	<0.001	PB	124	100	Sex
Mir et al. [26]	2019	Asian	Saudi Arabia	—	ARMS-PCR	7	0.152	PB	30	30	Sex
Pourmoshir et al. [27]	2020	Asian	Iran	—	ARMS-PCR	7	0.206	PB	153	153	Sex

Abbreviations: BRCA1, breast cancer type 1 susceptibility gene; BRCA2, breast cancer type 2 susceptibility gene; TNBC, triple-negative breast cancer; HB, hospital-based source of control; PB, population-based source of control; PCR, polymerase chain reaction; RFLP, restriction fragment length polymorphism; HRM, high-resolution melting; ARMS, amplification refractory mutation system; HWE, Hardy–Weinberg equilibrium.

**Table 2:** Genotype distributions of miR-423 rs6505162 polymorphism.

First author	Year	Ethnicity	Country	Sample size (cases/controls)	No. of cases			Allele frequencies of cases		No. of controls			Allele frequencies of controls	
					AA	AC	CC	A	C	AA	AC	CC	A	C
Kontorovich et al. [17]	2010	Caucasian	Israel	190/206	34	88	68	156	224	49	102	55	200	212
Smith et al. [18]	2012	Caucasian	Australia	179/174	60	95	24	215	143	42	80	52	164	184
Ma et al. [19]	2013	Asian	China	192/189	8	57	127	73	311	10	69	110	89	289
He et al. [20]	2015	Asian	China	450/450	16	142	292	174	726	22	129	299	173	727
Zhang et al. [21]	2015	Asian	China	382/189	20	131	231	171	593	10	69	110	89	289
Zhao et al. [22]	2015	Asian	China	114/189	5	30	79	40	188	10	69	110	89	289
Morales et al. [7]	2016	Caucasian	Chile	440/807	86	229	125	401	479	138	385	284	661	953
Saedi et al. [23]	2017	Asian	Iran	353/353	15	125	213	155	551	36	137	180	209	497
Tran Thi et al. [24]	2018	Asian	Vietnam	106/116	5	34	67	44	168	3	49	64	55	177
Mir et al. [25]	2018	Asian	Saudi Arabia	100/124	23	52	25	98	102	18	25	81	61	187
Mir et al. [26]	2019	Asian	Saudi Arabia	30/30	3	11	16	17	43	4	9	17	17	43
Pourmoshir et al. [27]	2020	Asian	Iran	153/153	59	46	48	164	142	67	63	23	197	109

Abbreviations: mir-423, microRNA-423.

**Table 3:** Methodological quality of studies included in the final analysis based on the Newcastle–Ottawa Scale for assessing the quality of case-control studies.

Study	Selection (score)				Comparability (score)	Exposure (score)			Total score^b^
	Adequate definition of patient cases	Representativeness of patient cases	Selection of controls	Definition of controls	Control for important factor or additional factor	Ascertainment of exposure (blinding)	Same method of ascertainment for participants	Non-response rate^a^	
Kontorovich et al. [17]	1	1	1	1	0	0	1	1	6
Smith et al. [18]	1	1	0	1	2	0	1	1	7
Ma et al. [19]	1	1	0	1	2	0	1	1	7
He et al. [20]	1	1	1	1	2	0	1	1	8
Zhang et al. [21]	1	1	1	1	2	0	1	1	8
Zhao et al. [22]	1	1	1	1	0	0	1	1	6
Morales et al. [7]	1	1	0	1	1	0	1	1	6
Saedi et al. [23]	1	1	0	1	1	0	1	1	6
Tran Thi et al. [24]	1	1	1	1	0	0	1	1	6
Mir et al. [25]	1	1	1	1	1	0	1	1	7
Mir et al. [26]	1	1	1	1	1	0	1	1	7
Pourmoshir et al. [27]	1	1	1	1	1	0	1	1	7

^a^When there was no significant difference in the response rate between both groups based on a chi-squared test (*P* > 0.05), one point was awarded. ^b^Total score was calculated by adding up the points awarded in each item.

**Table 4:** Overall meta-analysis of the association between breast cancer and miR-423 rs6505162 polymorphism.

Genetic model	OR [95 % CI]	Z (*P* value)	Heterogeneity of study design			Analysis model
			*χ*2	d*f* (*P* value)	*I* ^2^ (%)	
*Mir-423 rs6505162 in total population from 12 case control studies* [7, 17–27] *(2,689 cases and 2,980 controls)*						
Allelic model (C-allele vs. A-allele)	1.02 [0.81, 1.28]	0.19 (0.85)	73.30	11 (<0.001)	85	Random
Recessive model (CC vs. AC + AA)	0.99 [0.72, 1.38]	0.03 (0.97)	82.60	11 (<0.001)	87	Random
Dominant model (AA vs. AC + CC)	0.93 [0.72, 1.21]	0.52 (0.60)	21.40	11 (0.03)	49	Random
Homozygous model (CC vs. AA)	1.04 [0.66, 1.65]	0.17 (0.87)	54.28	11 (<0.001)	80	Random
Heterozygous model (AC vs. AA)	1.07 [0.90, 1.28]	0.76 (0.45)	11.49	11 (0.40)	4	Fixed

*Mir-423 rs6505162 in Asian population from 9 case-control studies* [19–27] *(1,880 cases and 1,793 controls)*						
Allelic model (C-allele vs. A-allele)	1.09 [0.82, 1.44]	0.58 (0.56)	47.22	8 (<0.001)	83	Random
Recessive model (CC vs. AC + AA)	1.10 [0.75, 1.61]	0.47 (0.64)	55.74	8 (<0.001)	86	Random
Dominant model (AA vs. AC + CC)	0.81 [0.63, 1.03]	1.72 (0.09)	11.91	8 (0.16)	33	Fixed
Homozygous model (CC vs. AA)	1.20 [0.69, 2.08]	0.64 (0.52)	29.58	8(<0.001)	73	Random
Heterozygous model (AC vs. AA)	1.20 [0.92, 1.56]	1.35 (0.18)	9.04	8(0.34)	11	Fixed

*Mir-423 rs6505162 in Chinese population from 4 case-control studies* [19–22] *(1,138 cases and 1,017 controls)*						
Allelic model (C-allele vs. A-allele)	1.12 [0.97, 1.30]	1.50 (0.13)	3.37	3 (0.34)	11	Fixed
Recessive model (CC vs. AC + AA)	1.13 [0.95, 1.35]	1.35 (0.18)	4.99	3 (0.17)	40	Fixed
Dominant model (AA vs. AC + CC)	0.81 [0.54, 1.22]	1.00 (0.32)	0.39	3 (0.94)	0	Fixed
Homozygous model (CC vs. AA)	1.29 [0.85, 1.95]	1.19 (0.24)	0.36	3 (0.95)	0	Fixed
Heterozygous model (AC vs. AA)	1.15 [0.75, 1.76]	0.62 (0.53)	1.10	3 (0.78)	0	Fixed

*Mir-423 rs6505162 in Caucasian population from 3 case-control studies* [7, 17, 18] *(809 cases and 1,187 controls)*						
Allelic model (C-allele vs. A-allele)	0.87 [0.58, 1.31]	0.66 (0.51)	16.19	2 (<0.001)	88	Random
Recessive model (CC vs. AC + AA)	0.75 [0.38, 1.48]	0.82 (0.41)	17.51	2 (<0.001)	89	Random
Dominant model (AA vs. AC + CC)	1.11 [0.74, 1.66]	0.49 (0.63)	5.80	2 (0.06)	66	Random
Homozygous model (CC vs. AA)	0.75 [0.33, 1.70]	0.70 (0.49)	16.17	2 (<0.001)	88	Random
Heterozygous model (AC vs. AA)	0.98 [0.77, 1.24]	0.20 (0.84)	1.25	2 (0.54)	0	Fixed

*Mir-423 rs6505162 in female population from 5 case-control studies* [18–21, 27] *(1,356 cases and 1,155 controls)*						
Allelic model (C-allele vs. A-allele)	1.05 [0.77, 1.42]	0.29 (0.78)	21.54	4 (<0.001)	81	Random
Recessive model (CC vs. AC + AA)	1.06 [0.66, 1.71]	0.24 (0.81)	27.48	4 (<0.001)	85	Random
Dominant model (AA vs. AC + CC)	1.00 [0.77, 1.30]	0.02 (0.99)	5.86	4 (0.21)	32	Fixed
Homozygous model (CC vs. AA)	1.08 [0.52, 2.27]	0.21 (0.83)	21.54	4 (<0.001)	81	Random
Heterozygous model (AC vs. AA)	0.95 [0.72, 1.26]	0.34 (0.73)	2.34	4 (0.67)	0	Fixed

Abbreviations: mir-423, microRNA-423; OR, odds ratios; 95% CI, 95% confidence interval.

## Data Availability

The data used to support the findings of this study are included within the article.

## Abbreviations

miR: MicroRNA
BC: Breast cancer
HWE: Hardy–Weinberg equilibrium
CNKI: Chinese National Knowledge Infrastructure
OR: Odds ratios
95% CI: 95% confidence interval
TNBC: Triple-negative breast cancer
HB: Hospital-based source of control
PB: Population-based source of control
PCR: Polymerase chain reaction
RFLP: Restriction fragment length polymorphism
HRM: High-resolution melting
ARMS: Amplification refractory mutation system.
